# Supplementary figures and images for: Cyclic mechanical stretch down-regulates cathelicidin antimicrobial peptide expression and activates a pro-inflammatory response in human bronchial epithelial cells
Source: PeerJ. 2015 Dec 7;3:e1483. doi: 10.7717/peerj.1483 (PMC4675098; doi:10.7717/peerj.1483)

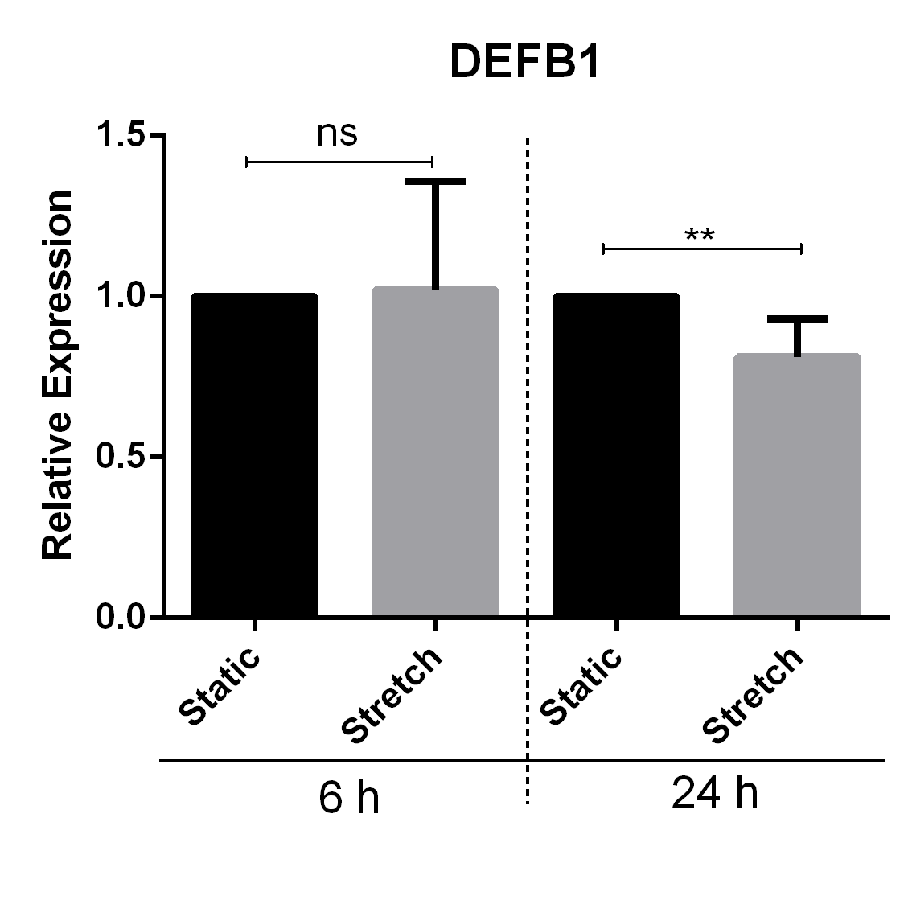

Supplement: Figure S1 — VA10 cells were subjected to cyclic stretch for 6 h and 24 h. The mRNA expression of DEFB1 was analyzed with q-RT PCR (n = 3, mean ± S.E. ). Relative expression levels (y-axis) in static cells were defined with an arbitrary value of ‘1’ and changes relative to this value in stretched samples are represented. (ns indicates non-significant; p < 0.01 = ∗∗). [file peerj-03-1483-s001.png]
