# Supplementary material for: Cyclic mechanical stretch down-regulates cathelicidin antimicrobial peptide expression and activates a pro-inflammatory response in human bronchial epithelial cells
Source: PeerJ. 2015 Dec 7;3:e1483. doi: 10.7717/peerj.1483 (PMC4675098; doi:10.7717/peerj.1483)
Supplement: Supplemental Information 2 [file peerj-03-1483-s002.docx]

|  |  |  |  |
| --- | --- | --- | --- |
| **Figure 1A** |  |  |  |
| **Sample** | **Mean Relative Expression (2-ddCt)** | **SEM (+/-)** | **p value** |
| **Static 6h** | 1 | 0 | - |
| **Stretch 6h** | 0,6383 | 0,0966 | 0,0038 |
| **Static 24h** | 1 | 0 | - |
| **Stretch 24h** | 0,62 | 0,06819 | 0,0001 |
|  |  |  |  |
| **Figure 2A** | **Mean Relative Expression (2-ddCt)** | **SEM (+/-)** | **p value** |
| **Static 6h** | 1 | 0 | - |
| **Stretch 6h** | 0,6383 | 0,0966 | 0,0038 |
| **Static + 1,25D3 6h** | 14,95 | 0,4657 | - |
| **Stretch + 1,25D3 6h** | 14,09 | 0,6645 | 0,3124 |
| **Static 24h** | 1 | 0 | - |
| **Stretch 24h** | 0,62 | 0,06819 | 0,0001 |
| **Static + 1,25D3 24h** | 53,04 | 3,392 | - |
| **Stretch + 1,25D3 24h** | 38,83 | 1,839 | 0,0132 |
| **Static 6h** | 1 | 0 | - |
| **Static + 1,25D3 6h** | 14,95 | 0,4657 | < 0,0001 |
|  |  |  |  |
| **Figure 2B** | **Mean Relative Expression (2-ddCt)** | **SEM (+/-)** | **p value** |
| **Static 6h** | 1 | 0 | - |
| **Stretch 6h** | 0,6383 | 0,0966 | 0,0038 |
| **Static + PBA 6h** | 3,255 | 0,2374 | - |
| **Stretch + PBA 6h** | 5,332 | 1,136 | 0,1039 |
| **Static 24h** | 1 | 0 | - |
| **Stretch 24h** | 0,62 | 0,06819 | 0,0001 |
| **Static + PBA 24h** | 13,20 | 0,5892 | - |
| **Stretch + PBA 24h** | 4,983 | 0,5191 | < 0,0001 |
| **Static 6h** | 1 | 0 | - |
| **Static + PBA 6h** | 3,255 | 0,2374 | < 0,0001 |
|  |  |  |  |
|  |  |  |  |
| **Figure 2C** | **Mean Relative Expression (2-ddCt)** | **SEM (+/-)** | **p value** |
| **Static 6h** | 1 | 0 | - |
| **Stretch 6h** | 0,6383 | 0,0966 | 0,0038 |
| **Static +1,25D3+PBA 6h** | 35,68 | 3,116 |  |
| **Stretch +1,25D3+PBA 6h** | 35,59 | 3,955 | 0,9866 |
| **Static 24h** | 1 | 0 | - |
| **Stretch 24h** | 0,62 | 0,06819 | 0,0001 |
| **Static +1,25D3+PBA 24h** | 207,3 | 6,170 | - |
| **Stretch +1,25D3+PBA 24h** | 113,2 | 5,999 | < 0,0001 |
|  |  |  |  |
| **Figure 2D** | **Mean Relative Expression (2-ddCt)** | **SEM (+/-)** | **p value** |
| **Static 6h** | 1 | 0 | - |
| **Stretch 6h** | 1,029 | 0,1870 | 0,8518 |
| **Static + 1,25D3 6h** | 72,32 | 5,724 | - |
| **Stretch + 1,25D3 6h** | 50,77 | 3,505 | 0,0184 |
| **Static 24h** | 1 | 0 | - |
| **Stretch 24h** | 0,5298 | 0,1049 | 0,0012 |
| **Static + 1,25D3 24h** | 42,65 | 4,721 | - |
| **Stretch + 1,25D3 24h** | 23,94 | 5,754 | 0,0772 |
|  |  |  |  |
| **Figure 3A** | **Mean Relative Expression (2-ddCt)** | **SEM (+/-)** | **p value** |
| **Static 6h** | 1 | 0 | - |
| **Stretch 6h** | 2,736 | 0,7463 | 0,0093 |
| **Static 24h** | 1 | 0 | - |
| **Stretch 24h** | 4,599 | 1,786 | 0,0179 |
|  |  |  |  |
| **Figure 3B** | **Mean Relative Expression (2-ddCt)** | **SEM (+/-)** | **p value** |
| **Static 6h** | 1 | 0 | - |
| **Stretch 6h** | 1,422 | 0,07805 | 0,0003 |
| **Static 24h** | 1 | 0 | - |
| **Stretch 24h** | 1,959 | 0,2504 | 0,0033 |
|  |  |  |  |
| **Figure 3C** | **Mean Relative Expression (2-ddCt)** | **SEM (+/-)** | **p value** |
| **Static 6h** | 1 | 0 | - |
| **Stretch 6h** | 0,1638 | 0,006888 | < 0,0001 |
| **Static 24h** | 1 | 0 | - |
| **Stretch 24h** | 0,7568 | 0,03817 | < 0,0001 |
|  |  |  |  |
| **Figure 3D** | **Mean Relative Expression (2-ddCt)** | **SEM (+/-)** | **p value** |
| **Static 6h** | 1 | 0 | - |
| **Stretch 6h** | 1,107 | 0,09453 | 0,2842 |
| **Static 24h** | 1 | 0 | - |
| **Stretch 24h** | 0,7033 | 0,0353 | < 0,0001 |
|  |  |  |  |
| **Figure 3E** | **Mean (pg/ml)** | **SEM (+/-)** | **p value** |
| **Static** | 111,6 | 5,064 | - |
| **Stretch** | 190,1 | 13,42 | 0,0007 |
|  |  |  |  |
| **Figure 3F** | **Mean (pg/ml)** | **SEM (+/-)** | **p value** |
| **Static** | 608,9 | 27.25 | - |
| **Stretch** | 445.9 | 32.06 | 0,0018 |
|  |  |  |  |
| **Figure 3G** | **Mean (% positive ROS)** | **SEM (+/-)** | **p value** |
| **Static** | 52,95 | 1,350 | - |
| **Stretch** | 80,80 | 3,616 | 0,0071 |
|  |  |  |  |
|  |  |  |  |
| **Figure 4A** | **Mean Relative Expression (2-ddCt)** | **SEM (+/-)** | **p value** |
| **Static 6h** | 1 | 0 | - |
| **Stretch 6h** | 0,9602 | 0,1420 | 0,7850 |
| **Static 24h** | 1 | 0 | - |
| **Stretch 24h** | 1,050 | 0,1321 | 0,6849 |
|  |  |  |  |
| **Figure 4B** | **Mean Relative Expression (2-ddCt)** | **SEM (+/-)** | **p value** |
| **Static 6h** | 1 | 0 | - |
| **Stretch 6h** | 1,244 | 0,1371 | 0,1051 |
| **Static 24h** | 1 | 0 | - |
| **Stretch 24h** | 1,541 | 0,1277 | 0,0017 |
|  |  |  |  |
| **Figure 4C** | **Mean Relative Expression (2-ddCt)** | **SEM (+/-)** | **p value** |
| **Static 6h** | 1 | 0 | - |
| **Stretch 6h** | 0,7919 | 0,008021 | **< 0.0001** |
| **Static 24h** | 1 | 0 | - |
| **Stretch 24h** | 0,6856 | 0,1216 | 0,0114 |
|  |  |  |  |
| **Figure 4D** | **Mean Relative Expression (2-ddCt)** | **SEM (+/-)** | **p value** |
| **Static 6h** | 1 | 0 | - |
| **Stretch 6h** | 1,332 | 0,1619 | 0,0675 |
| **Static 24h** | 1 | 0 | - |
| **Stretch 24h** | 0,9555 | 0,08754 | 0,622 |
|  |  |  |  |
| **Figure 4E** | **Mean Relative Expression (2-ddCt)** | **SEM (+/-)** | **p value** |
| **Static 6h** | 1 | 0 | - |
| **Stretch 6h** | 0,7595 | 0,07465 | 0,006 |
| **Static 24h** | 1 | 0 | - |
| **Stretch 24h** | 0,9113 | 0,1970 | 0,662 |
|  |  |  |  |
| **Figure 4F** | **Mean Relative Expression (2-ddCt)** | **SEM (+/-)** | **p value** |
| **Static 6h** | 1 | 0 | - |
| **Stretch 6h** | 0,9500 | 0,1356 | 0,7198 |
| **Static 24h** | 1 | 0 | - |
| **Stretch 24h** | 0,9961 | 0,09114 | 0,9665 |
|  |  |  |  |
| **Figure 4G** | **Mean Relative Expression (2-ddCt)** | **SEM (+/-)** | **p value** |
| **Static 6h** | 1 | 0 | - |
| **Stretch 6h** | 0,7003 | 0,1198 | 0,0133 |
| **Static 24h** | 1 | 0 | - |
| **Stretch 24h** | 1,024 | 0,1890 | 0,8924 |
|  |  |  |  |
| **Figure 4H** | **Mean Relative Expression (2-ddCt)** | **SEM (+/-)** | **p value** |
| **Static 6h** | 1 | 0 | - |
| **Stretch 6h** | 0,9111 | 0,1029 | 0,4079 |
| **Static 24h** | 1 | 0 | - |
| **Stretch 24h** | 0,8017 | 0,03118 | < 0,0001 |
|  |  |  |  |
| **Figure 5A** | **Mean Relative Expression (2-ddCt)** | **SEM (+/-)** | **p value** |
| **Static 6h** | 1 | 0 | - |
| **Stretch 6h** | 1,982 | 0,1393 | < 0,0001 |
| **Static + 1,25D3 6h** | 1,377 | 0,09657 |  |
| **Stretch + 1,25D3 6h** | 2,544 | 0,2948 | 0,0022 |
| **Static 24h** | 1 | 0 | - |
| **Stretch 24h** | 1,574 | 0,4109 | 0,0703 |
| **Static + 1,25D3 24h** | 1,462 | 0,1778 | - |
| **Stretch + 1,25D3 24h** | 2,246 | 0,2400 | 0,0251 |
| **Static 6h** | 1 | 0 | - |
| **Static + 1,25D3 6h** | 1,377 | 0,09657 | 0,0029 |
| **Stretch 6h** | 1,982 | 0,1393 | - |
| **Stretch + 1,25D3 6h** | 2,544 | 0,2948 | 0,0894 |
| **Static 24h** | 1 | 0 | - |
| **Static + 1,25D3 24h** | 1,462 | 0,1778 | 0,0265 |
|  |  |  |  |
|  |  |  |  |
| **Figure 5B** | **Mean Relative Expression (2-ddCt)** | **SEM (+/-)** | **p value** |
| **Static 6h** | 1 | 0 | - |
| **Stretch 6h** | 2,736 | 0,7463 | 0,0093 |
| **Static + PBA 6h** | 1,622 | 0,04712 | - |
| **Stretch + PBA 6h** | 9,311 | 1,300 | 0,0169 |
| **Static 24h** | 1 | 0 | - |
| **Stretch 24h** | 4,599 | 1,786 | 0,0179 |
| **Static + PBA 24h** | 4,930 | 0,6048 | - |
| **Stretch + PBA 24h** | 4,285 | 0,3246 | 0,4429 |
| **Static 6h** | 1 | 0 | - |
| **Static + PBA 6h** | 1,622 | 0,04712 | < 0,0001 |
| **Stretch 6h** | 2,736 | 0,7463 | - |
| **Stretch + PBA 6h** | 9,311 | 1,300 | 0,0107 |
| **Static 24h** | 1 | 0 | - |
| **Static + PBA 24h** | 4,930 | 0,6048 | < 0.0001 |
|  |  |  |  |
|  |  |  |  |
| **Figure S1** | **Mean Relative Expression (2-ddCt)** | **SEM (+/-)** | **p value** |
| **Static 6h** | 1 | 0 | - |
| **Stretch 6h** | 1,021 | 0,1372 | 0,8801 |
| **Static 24h** | 1 | 0 | - |
| **Stretch 24h** | 0,8121 | 0,05913 | 0,0038 |
